# Size-Dependent Photodynamic Activity of Gold Nanoparticles Conjugate of Water Soluble Purpurin-18-*N*-Methyl-*D*-Glucamine

**DOI:** 10.1155/2013/720579

**Published:** 2012-12-30

**Authors:** Byambajav Lkhagvadulam, Jung Hwa Kim, Il Yoon, Young Key Shim

**Affiliations:** PDT Research Institute, School of Nano System Engineering, Inje University, Gimhae 621-749, Republic of Korea

## Abstract

Gold nanoparticles (GNPs) conjugates of water soluble ionic photosensitizer (PS), purpurin-18-*N*-methyl-*D*-glucamine (Pu-18-NMGA), were synthesized using various molar ratios between HAuCl_4_ and Pu-18-NMGA without adding any particular reducing agents and surfactants. The PS-GNPs conjugates showed long wavelength absorption of range 702–762 nm, and their different shapes and diameters depend on the molar ratios used in the synthesis. *In vitro* anticancer efficacy of the PS-GNPs conjugates was investigated by MTT assay against A549 cells, resulting in higher photodynamic activity than that of the free Pu-18-NMGA. Among the PS-GNPs conjugates, the GNPs conjugate from the molar ratio of 1 : 2 (Au(III): Pu-18-NMGA) exhibits the highest photodynamic activity corresponding to bigger size (~60 nm) of the GNPs conjugate which could efficiently transport the PS into the cells than that of smaller size of the GNPs conjugate.

## 1. Introduction

Photodynamic therapy (PDT) is a promising noninvasive cancer treatment by using a combination of photosensitizer (PS), light, and oxygen [1–3]. For excellent photodynamic activity, PS should be penetrated into the tumor cells sufficiently [4]. Most of PSs are hydrophobic and thus locate preferentially in the lipid bilayers of organelle membranes in cancer cells. However, the hydrophobic nature of PSs makes them insoluble under physiological conditions and hinders to reach the accumulation in the tumor sites [5]. Therefore, for both hydrophobic and hydrophilic (amphiphilic) environments of PSs, introduction of water soluble PS with suitable carrier is a one potential method [6–8]. On the other hand, highly water soluble (hydrophilic) PSs allow poor cellular uptake based on a short pharmacological half-life which may have limit to penetrate through the tissue and cell membranes [9–11]. 

Nanoparticles (NPs) [12–14] are promising carrier system of PSs that could be protected from being uptaken by the reticuloendothelial system and extended the circulation time of NPs in the blood, and, finally, preferentially accumulated in tumor sites through the so-called “enhanced permeability and retention (EPR)” effect [15–17]. Among the NPs, gold nanoparticles (GNPs) are highly efficient PDT drug delivery platform with good advantages based on their chemical inertness and minimum toxicity that has potential applications in biomedicine such as photothermal therapy (PTT) [18–21] of cancer, gene and drug delivery, biological imaging, and biosensing [22–26]. In addition, GNPs have large surface-to-volume ratios and easy tuning of the NPs size, resulting in penetration into tumor cells and intracellular localization at endosomes/lysosomes of the cells, and finally targeting at mitochondrial of cancer cells induces apoptosis to destroy the cancer cells [27–31]. It is noted that the size of the GNPs plays a big role in their uptake at the cellular level leading to different PDT activity. However, to the best of our knowledge, there are few reports for relationship between GNPs and its size effect on photodynamic activity [28].

**Figure 1:**
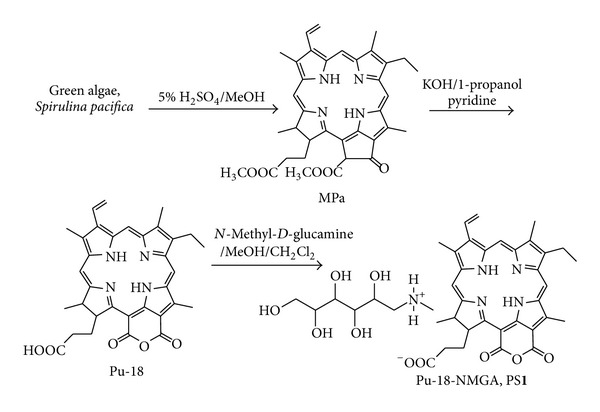
Synthetic method of *N*-methyl-*D*-glucamine salt of purpurin-18 (Pu-18-NMGA, PS**1**).

Previously, we developed new synthesis of PS-GNPs conjugate using water soluble ionic purpurin-18-*N*-methyl-*D*-glucamine (Pu-18-NMGA, PS**1**, Figure 1) and this conjugate showed better *in vitro* anticancer efficacy than that of free PS**1** against A549 lung cancer cells [32]. 

In this paper, we have synthesized various sizes of PS-GNPs conjugates using a simple single-step synthesis from different molar ratios of HAuCl_4_/PS**1** without adding any particular reducing agents and surfactants, and showed size effect allowed different photodynamic activity results of the conjugates as an important factor for PDT. We evaluated *in vitro* anticancer efficacy of the PS-GNPs conjugates against A549 cells using MTT assay. 

## 2. Materials and Methods

### 2.1. Materials

All reagents were purchased from Aldrich and used without further purification. All aqueous solutions were made using triply distilled water. All reactions were monitored by thin-layer chromatography (TLC) using Merck 60 silica gel F254 precoated (0.2 mm thickness) glass-backed sheets. Silica gel 60A (230–400 mesh, Merck) was used for column chromatography. The ^1^H NMR spectra were obtained using a Varian spectrometer (500 MHz) at Biohealth Products Research Center (BPRC) at Inje University. The chemical shifts (*δ*) are given in parts per million (ppm) relative to tetramethylsilane (TMS, 0 ppm). High-resolution fast atom bombardment mass (HRFABMS) spectra were obtained with a Jeol JMS700 high-resolution mass spectrometer at the Daegu center of KBSI, Kyungpook National University, Korea.

The PS**1** and PS-GNPs conjugates **2a**–**2e** were characterized by a combination analysis of ^1^H-NMR and UV-vis spectroscopies, transmission electron microscopy (TEM), and infrared (IR) spectroscopy. UV-vis absorption spectra were recorded using a SCINCO S-3100 UV-vis spectrophotometer using 1 cm quartz cuvette. TEM images were performed on a JEOL, JEM 2011. A typical sample for TEM was prepared by drying of a drop of the solution at room temperature on a carbon-coated copper grid. IR spectra were measured on a Varian-640 FT-IR spectrometer.

### 2.2. Synthesis

Methyl pheophorbide-a (MPa) [33], purpurin-18 (Pu-18) [34], and *N*-methyl-*D*-glucamine salt of purpurin-18 (Pu-18-NMGA, PS**1**) [32] were prepared according to the procedures in literature, and all analytical data are identical with those in the literatures.

***N*-Methyl-*D*-Glucamine Salt of Purpurin-18 (Pu-18-NMGA, PS1) [32]**

To a solution of Pu-18 (56.4 mg, 0.1 mmol) in MeOH/CH_2_Cl_2_ (3 : 1, 10 mL), a solution of NMGA (39.0 mg, 0.2 mmol) in MeOH/water (1 : 2, 20 mL) was added and the mixture was stirred for 4 h. The organic solvents were evaporated under vacuum and the resulting aqueous solution was filtered through a membrane (20 *μ*m) and freeze-dried to give PS**1**. Yield: 68.0 mg (87%). UV-vis (water): *λ*, nm (log *ε*) 282 (0.34), 379 (0.55), 388 (0.58), 405 (0.61), 501 (0.13), 561 (0.11), 652 (0.28), 702 (0.21). ^1^H NMR (500 MHz, CD_3_OD, 25°C, TMS) *δ*, ppm 9.68 (1H, s, H-5), 9.39 (1H, s, H-10), 8.95 (1H, s, H-20), 8.69 (2H, s, H-NH gluc), 7.80 (1H, m, H-3^1^), 6.24 and 6.12 (2H, dd, H-3^2^), 5.07 (1H, m, H-18), 4.49 (1H, m, H-17), 4.05 (2H, m, H-1 gluc), 3.83 (4H, m, OH-2,3,4,5 gluc), 3.69 (4H, m, H-2,3,4,5 gluc), 3.63 (3H, s, H-12), 3.48 (2H, m, H-8^1^), 3.21 (3H, s, H-2^1^), 3.18 (1H, m, OH-1 gluc), 3.07 (2H, dd, H-6 gluc), 3.03 (3H, s, H-7^1^), 2.74 (3H, s, H-7 gluc), 2.46 (2H, m, H-17^1^), 2.26 (3H, m, H-17^2^), 1.95 (3H, m, H-18^1^), 1.83 (3H, d, H-8^2^), 1.69 (1H, br s, NH), 1.32 (1H, br s, NH). HRFABMS: calcd for C_40_H_50_N_5_O_10_ ([M + H]^+^) 760.3558, found 760.3554.

**Synthesis of PS-GNPs Conjugates 2a–2e**

The GNPs were synthesized according to the seed growth method [25] with some modifications. PS-GNPs conjugates **2a**–**2e** were synthesized from different molar ratios between Au(III) and PS**1** through reduction of chloroauric acid (HAuCl_4_) with no use of reducing agent or surfactant.

**Preparation of Seed Solution of PS-GNPs Conjugate**

0.002 M solution of PS**1** (5 mL) was mixed with 0.001 M HAuCl_4_ (2.5 mL) in a 50 mL flat bottom flask and was stirred at room temperature for 2 h. The solution color was changed from yellow to greenish black, and then the solution was stored at room temperature.

**Growth of PS-GNPs Conjugate**

0.001 M solution of HAuCl_4_ (25 mL) is added to suitable concentration (for various molar ratios between Au(III) and PS**1**) of PS**1** solution (25 mL) in a 250 mL flat bottom flask (the color of solution was changed from yellow to green). Then 0.005 M AgNO_3_ solution (1 mL) was added to the mixture. To this mixture, the seed solution (100 *μ*L) was added to the center of the solution. Then the flask was never moved, so that the seed started to grow in the growth solution. After few minutes, the each PS-GNPs conjugate was obtained and washed with water for several times and was centrifuged at 10,000 rpm for 10 min and resuspended in water. Selected data for **2a**: UV-vis (water): *λ*, nm (log*ε*) 275 (0.36), 373 (0.43), 388 (0.46), 393 (0.48), 524 (0.21), 560 (0.25), 672 (0.23), 714 (0.35). ^1^H NMR (500 MHz, CD_3_OD, 25°C, TMS) *δ*, ppm 8.91 (2H, s, H-5 and H-1 gluca), 8.74 (1H, s, H-10), 8.64 (3H, s, H-20 and H-NH gluc), 7.82 (1H, m, H-3^1^), 6.21and 6.12 (2H, dd, H-3^2^), 4.95 (1H, m, H-18), 4.41 (1H, m, H-17), 3.62 (3H, s, H-12^1^), 3.41 (2H, d, H-8^1^), 3.21 (3H, s, H-2^1^), 3.16 (2H, d, H-6 gluc), 2.82 (6H, s, H-7 gluc and 7^1^), 2.63 (2H, m, H-17^1^), 2.30 (2H, m, H-17^2^), 1.75 (3H, d, H-18^1^), 1.33 (3H, m, H-8^1^), 0.88 (1H, br s, NH), 0.08 (1H, br s, NH). ATR IR (cm^−1^): 3400 (w, stretching –NH_2_^+^–), 1760 (s, stretching C=O), 1525 (s), 1300–1100 (stretching, bending C=O).

### 2.3. Cell Culture and Photo Irradiation

A549 human lung carcinoma cell lines were obtained from the cell line bank at Seoul National University's cancer research center and were grown in medium RPMI-1640 (Sigma-Aldrich) with 10% fetal bovine serum, glutamine, penicillin, and streptomycin at 37°C in humidified atmosphere of 5% CO_2_ in air. Phosphate-buffered saline (PBS) (Sigma-Aldrich), microscope (Olympus, CK40-32 PH), ELISA-reader (BioTek, SynergyHT), trypsin-EDTA, solution and incubator (37°C, 5% CO_2_) were used. The PDT was carried out using a diode laser generator apparatus (BioSpec LED, Russia) equipped with a halogen lamp, a bandpass filter (640–710 nm), and a fiber optics bundle. The duration of light irradiation, under PDT treatment, is calculated taking into account the empirically found effective dose of light energy in J*·*cm^2^.

### 2.4. MTT Assay and Cell Viability

A549 Cells (1 × 10^5^ cells/well) in 100 *μ*L of the mixed medium were placed in a 96-well plate and incubated for 48 h (37°C, 5% CO_2_). The medium was removed and the cultures were washed 3 times with physiologic saline. And the Pu-18-NMGA PS**1** (0.8–25 *μ*g/mL) or corresponding amount of the PS-GNPs conjugates **2a**–**2e** (constant amount of the PS**1**) in 100 *μ*L of the mixed medium was added in each well. 24 h later, the Pu-18-NMGA PS**1** or each PS-GNPs conjugate solution was discarded, and the cultures were washed 3 times with physiological saline and then medium (100 *μ*L/well) was added. The cultures were then subjected to the irradiation (2 J·cm^−2^) at the distance of 20 cm for 15 min, followed by an 3-(4,5-dimethylthiazole-2-yl)-2,5-biphenyl tetrazolium bromide (MTT) assay to evaluate their sensitivity to PDT. For the MTT assay, MTT solution (10 *μ*L) was added to each cell-culture well and cultured in the incubator for 3 h. Detergent solution (TACS, Trevigen, 200 *μ*L) was added to the culture, shaken for 10 min, and the absorbance was measured with an ELISA reader at 570 nm. Measurements were performed 3 h, 24 h, and 48 h incubation time after the irradiation, respectively. Each group consisted of 3 wells.

## 3. Results and Discussion

### 3.1. Preparation of Gold Nanoparticles Conjugates

The commonly used synthetic way of GNPs is a reduction method of Au(III) salt (usually from HAuCl_4_) using sodium citrate in water [35]. In this method, sodium citrate has a double role as a weak reducing agent as well as a capping agent that stabilizes the NPs. The particle size is controlled by a ratio between citrate and AuCl_4_^−^ ions. Higher concentration of citrate afforded smaller particle size [35].

**Figure 2:**
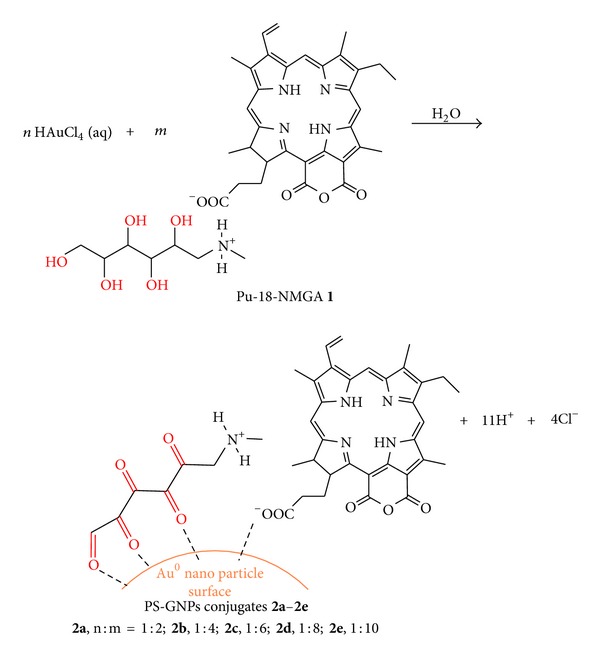
Synthetic method of PS-GNPs conjugates **2a**–**2e **with various molar ratios between gold and Pu-18-NMGA (*n* and *m* are molar ratios).

**Figure 3:**
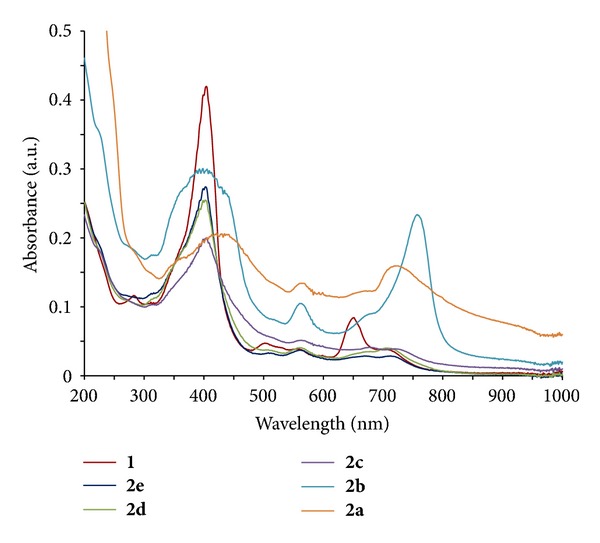
UV-vis spectra of (a) PS**1** and PS-GNPs conjugates **2a**–**2e **with various molar ratios between gold and Pu-18-NMGA in water (**2a**, Au:Pu-18-NMGA = 1 : 2; **2b**, 1 : 4; **2c**, 1 : 6; **2d**, 1 : 8; **2e**, 1 : 10).

However, in this work, we used hydrophilic PS**1** and Au(III) without any additional reducing agents and surfactants [32]. The hydroxyl groups of NMGA in PS**1** have important roles as a reducing agent as well as a stabilizer through the electrically charged functional groups (i.e., carboxylate and amine groups) in forming the PS-GNPs conjugates [9]. PS**1** was obtained from the carboxyl group of purpurin-18 (Pu-18) and the amine group of NMGA by simple and effective method (Figure 1). Pu-18 was synthesized from a conversion of methyl pheophorbide *a *(MPa) by air oxidation in *n*-propanol with KOH [34]. MPa was obtained from *Spirulina pacifica *algae by the procedure reported by Smith et al. [33]. PS-GNPs conjugates were prepared from the reaction of different molar ratios between Au(III) and PS**1** (**2a**, 1 : 2; **2b**, 1 : 4; **2c**, 1 : 6; **2d**, 1 : 8; **2e**, 1 : 10) in water to afford different particle sizes (Figure 2). The structures of the water soluble PS**1** and the PS-GNPs conjugates were confirmed by ^1^H-NMR spectroscopy, mass spectrometry, and UV-vis spectroscopy (Figure 3). 

The water soluble PS**1** acts not only as a reducing agent, but also as a capping agent in the reduction of HAuCl_4_ for synthesis of PS-GNPs conjugates. The formation of PS-GNPs conjugates is stable in the aqueous solution due to the adsorption of oxidized PS**1** on the surface of the GNPs through a strong coordinate-covalent bond between carboxylate on PS**1** and gold metal. So the binding strength of PS**1** on the GNPs surface is enough to allow accumulation of PS**1** in culture medium or *in vivo* [8, 24, 36, 37]. Therefore, a large amount of water soluble PS was generally used in order to get stable GNPs. Hence, we have used five different concentration ratios between Au(III) and PS**1** in order to find suitable concentration ratio that gives optimal size of the PS-GNPs conjugates for best photodynamic activity result.

### 3.2. UV-Vis Spectroscopic Investigation and Size Analysis by TEM Images

Figure 3 shows the UV-vis absorption spectra of the PS-GNPs conjugates **2a**–**2e** in water. In each conjugate, typical plasmon resonance band of the GNPs was appeared at 506–525 nm, respectively [24]. In **2a**–**2c**, the longest wavelength absorption (*λ*_max⁡_) is longer (719–762 nm) than that of PS**1** (702 nm), while *λ*_max⁡_ of **2d**–**2e** is the same with that of PS**1 **(Table 1). Among the conjugates, **2b** showed the longest wavelength absorption at 762 nm. In **2a**–**2b**, the Soret band at about 330–450 nm was broadened, indicating the formation of stacking structure of the chlorin ring on the gold surfaces [25]. 

**Figure 4:**
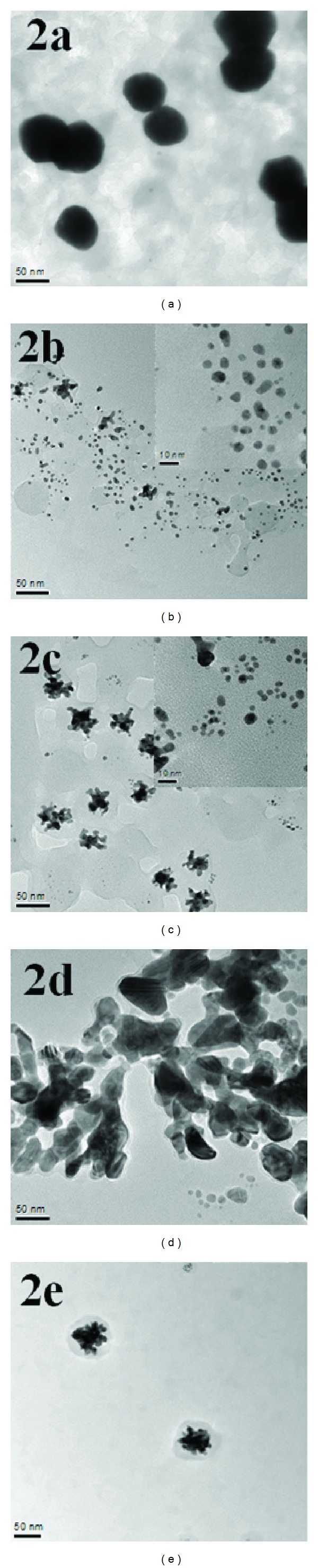
TEM images of the PS-GNPs conjugates **2a**–**2e**. The scale bars are 50 nm and the inset scale bars are 10 nm.

Figure 4 shows the typical TEM images of the PS-GNPs conjugates **2a**–**2e** prepared by using different concentration ratios between Au(III) and PS**1**. The images of the conjugates are different from each other in size and shape corresponding to the different molar ratios used in the preparation of the conjugates (Table 2). In **2a**, when molar ratio was 1 : 2 for Au(III) :  PS**1**, the GNPs are mainly peanut-shaped nanocrystals in water. And some spheres have diameters around 60 nm and are well dispersed with no aggregation between the GNPs in water. In **2b**, when molar ratio was 1 : 4, the GNPs are nanospheres have diameters around 5–11 nm. And some GNPs are closely placed each other and have a chain-like appearance with branching. In **2c**, when molar ratio was 1 : 6, the GNPs are nanospheres have diameters around 5–10 nm. However, some GNPs are aggregated together to form many bundles of GNPs, resulting in bigger diameters around 27–44 nm. In **2d**, when molar ratio was 1 : 8, the GNPs are mainly aggregated bundles and shape was not spheres with length around 50–90 nm and width around 25–50 nm. And yield of the GNPs was low and some aggregated GNPs have size around 200 nm. In **2e**, when molar ratio was 1 : 10, the GNPs are mainly aggregated and yield of the GNPs was very low, and some aggregated bundles of GNPs were around 50–70 nm size. When relatively lower molar ratio of PS**1** (2 or 4) made stable GNPs conjugate, however, higher molar ratio allowed unstable GNPs conjugate and remains continuous aggregation [35]. Consequently, the molar ratio between Au(III) and PS**1** is an important driving force to control GNPs size, shape, and aggregation degree of the GNPs in aqueous media.

Based on the UV-vis spectra and TEM images, there is a good relationship between absorption intensity and particle size. Higher absorption intensity of the conjugate corresponds to bigger particle size. In **2a**, absorption intensity at over than 450 nm ranges is the highest among all the conjugates, which corresponds to the biggest size (about 60 nm) in the conjugates. 

Compound **2b** shows the longest wavelength absorption at 762 nm which is included in NIR wavelength region (PTT therapeutic window, 750–1100 nm), so there is a potential for using PTT. We are considering that the GNPs conjugate for a combination (synergy effect) therapy of PDT and PTT [38, 39].

### 3.3. Photodynamic Activity and Size Effect by *In Vitro*

*In vitro* activity of the GNPs conjugates was evaluated by comparison with PS**1** against A549 human lung adenocarcinoma cells at 0.8, 1.6, 3.2, 6.1, 12.5, and 25 *μ*g/mL. In this case, PS**1** was dissolved in a mixed solvent of ethanol and water (1 : 1 volume ratio) and conjugates **2a**–**2e** were resuspended in water. The dark cytotoxicity and phototoxicity of **2a**–**2e** and PS**1** were measured by MTT assay at 3 h, 24 h, and 48 h incubation times, respectively.

**Figure 5:**
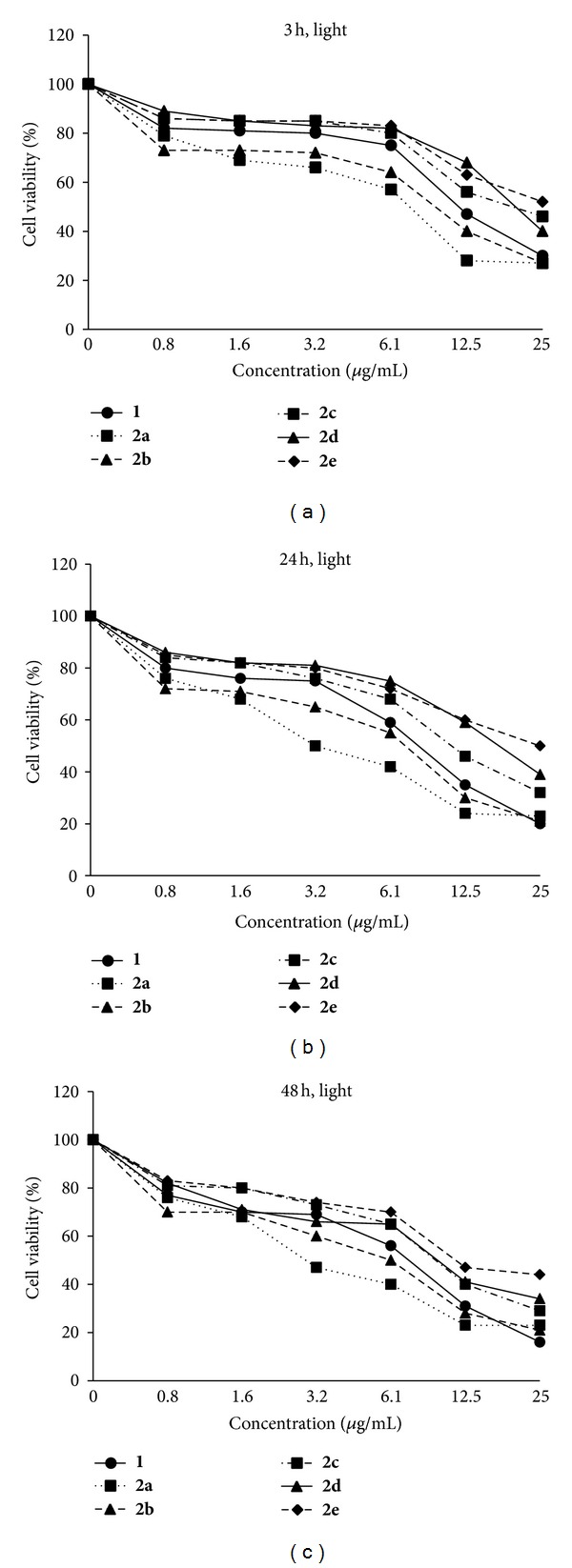
Cell viability (%) of PS**1** (0–25 *μ*g/mL) and the PS-GNPs conjugates **2a**–**2e** against A549 cells by exposure to an irradiation after 3 h, 24 h, and 48 h incubation times at 670–710 nm (2 J*·*cm^−2^) for 15 min.

**Figure 6:**
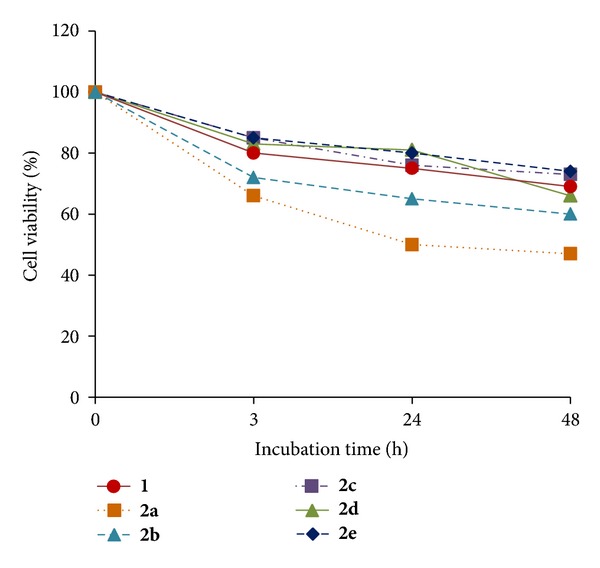
Comparative cell viability (%) of PS**1** (3.2 *μ*g/mL) and the PS-GNPs conjugates **2a**–**2e** against A549 cells by photo irradiation after 3 h, 24 h, and 48 h incubation times.

In all the compounds, upon photo irradiation, the cell viability was decreased corresponding to the increased incubation time after PDT as well as increased concentration (Figure 5), for example, at 48 h incubation and 3.2 *μ*g/mL, 80% at 3 h, 75% at 24 h, and 69% for PS**1**, and 66% at 3 h, 50% at 24 h, and 47% for **2a** (Figure 6), respectively. 

**Figure 7:**
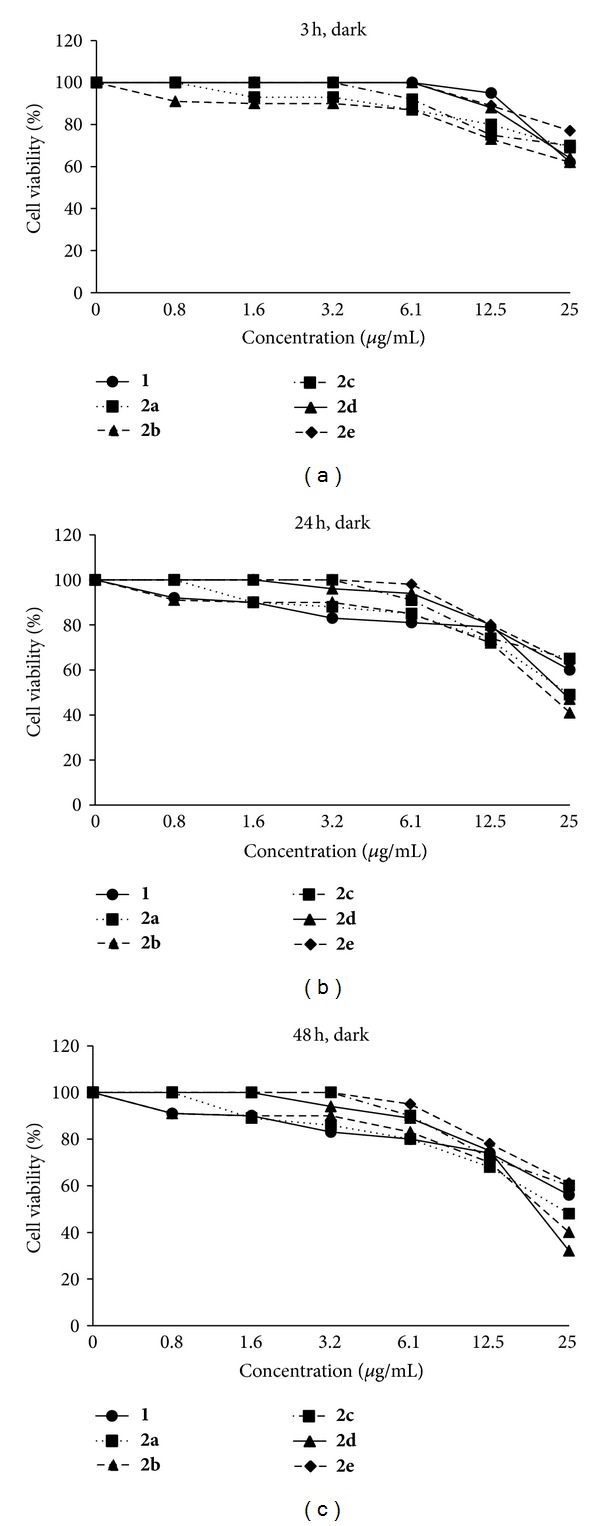
Cell viability (%) of PS**1** (0–25 *μ*g/mL) and the PS-GNPs conjugates **2a**–**2e** against A549 cells without photo irradiation (dark cytotoxicity) after 3 h, 24 h, and 48 h incubation times.

Dark cytotoxicity of PS**1** and conjugates **2a**–**2e** is shown in Figure 7. At highest concentration (25 *μ*g/mL) with 48 h incubation time, all compounds showed high dark cytotoxicity (cell viability 32–61%).

PS**1** showed slightly higher photocytotoxicity (IC_50_, 10.5 *μ*g/mL = 14 *μ*M at 24 h incubation time) than that of the purpurin-18-choline derivative (IC_50_, 15 *μ*M at 24 h incubation time) that has been previously reported by us [25]. Conjugates **2a** and **2b** showed higher photocytotoxicity than that of PS**1**. At high concentration (e.g., at 25 *μ*g/mL), **2a** showed higher dark cytotoxicity (69% at 3 h, 49% at 24 h, and 48% at 48 h incubation time, Figure 7) as compared to PS**1** (62% at 3 h, 60% at 24 h, and 56% at 48 h incubation time), which might be attributed to large amount of PS**1** molecules on the GNPs surface in **2a**. However, conjugates **2c**–**2e** showed lower photocytotoxicity than that of PS**1**. This result demonstrates that photodynamic activity significantly depends on size and aggregation degree of the GNPs. For example, in **2a** and **2b** there is no aggregation between each other and **2a** has about 60 nm size, while in **2c**–**2e** there are some aggregated bundles of the GNPs with small size. Chithrani et al. [29] studied a relationship between particles size (14–100 nm) and cellular uptake of the GNPs in HeLa cells, in which the maximum uptake was occurred at a size of 50 nm. Jiang et al. [28] have reported that cellular uptake strongly depends on the size of the GNPs, in which the GNPs having 2–100 nm size range were coated with Herceptin and were evaluated for cell internalization against breast cancer cell lines by the ErbB2 receptor. The most efficient cellular uptake was observed with particles range of 20–50 nm. Apoptosis was also enhanced by the GNPs having 40–50 nm size [28]. From the high dark cytotoxicity at high concentration, we confirmed that the PS-GNPs conjugate **2a** and **2b** showed better photodynamic activity at low concentration (3.2 *μ*g/mL) having low dark cytotoxicity (Figure 7). 

In addition, **2a** and **2b** have higher absorbance at irradiated wavelength range, which allowed good photodynamic activity results. In **2c**–**2e,** absorption intensity was lower than that of PS**1**, resulting in lower photocytotoxicity as compared to PS**1**.  Table 3 shows the IC_50_ values for PS**1** and its PS-GNPs conjugates **2a**–**2e**. At 48 h incubation time, **2a** and **2b** showed better IC_50_ value, 4.32 and 6.38 *μ*g/mL, respectively, as compared to PS**1** (8.72 *μ*g/mL). Therefore, as we pointed out above, photodynamic *in vitro *activity of synthesized PS-GNPs conjugates (**2a** and **2b**) is much higher than that of the free PS**1**. This result indicates that optimal size and well-dispersed nanoparticles are important for photodynamic effect in aqueous media. Especially, bigger size (~60 nm) of nanoparticles **2a** could be useful to transport more chlorine molecules into the cancer cells by endocytosis [28, 29, 40]. 

## 4. Conclusions

In summary, a simple single-step synthesis of PS-GNPs conjugates from different molar ratios of Au(III)/water soluble ionic PS**1** (purpurin-18-*N*-methyl-*D*-glucamine) has been studied without adding any particular reducing agents and surfactants. *In vitro *anticancer efficacy of the PS-GNPs conjugates against A549 lung cancer cell lines was evaluated. We revealed that PDT *in vitro* activity of synthesized PS-GNPs conjugates was higher as compared to free PS**1** because of good transport of the PS into the cells by using size effect. Conjugate **2a **based on molar ratio between HAuCl_4_ and PS was 1 : 2 that exhibits best PDT efficiency than other conjugates having different molar ratios. This result could be useful for synthesis of new PS and PS-GNPs conjugates having different size as well as for developing good relationship between PDT activity and size effect of GNPs in aqueous media.

## Figures and Tables

**Table 1:** Absorption properties of PS**1** and the PS-GNPs conjugates **2a**–**2e**.

Compound	Absorption *λ*_max⁡_ (nm) (log *ε*)	
	Soret	Qy
**1**	435 (0.64)	702 (0.67)
**2a**	440 (0.34)	724 (0.28)
**2b**	434 (0.69)	762 (0.77)
**2c**	435 (0.62)	719 (0.34)
**2d**	434 (0.69)	702 (0.50)
**2e**	434 (0.69)	702 (0.44)

**Table 2:** Summary of TEM images of the PS-GNPs conjugates **2a**–**2e**.

Compound	Shape	Diameter (nm)	Dispersion
**2a**	Sphere, peanut	∼60	GNPs are well dispersed and showed no aggregation.
**2b**	Sphere, chain-like appearance with branching	5–11	GNPs are well dispersed and showed no aggregation.
**2c**	Sphere	5–11	Some aggregated GNPs have diameter sizes of 27–44 nm.
**2d**	Not sphere	—	GNPs have a lot of aggregation.
**2e**	Not sphere	—	Very few aggregated GNPs have sizes of 50–70 nm.

**Table 3:** IC_50_ (*μ*g/mL) values of PS**1** and **2a**–**2e** against A549 cells at various incubation times. IC_50_ values were determined by MTT assay at 3 h, 24 h and 48 h incubation after photo irradiation.

Compound	3 h	24 h	48 h
**1**	14.8	10.5	8.72
**2a**	7.14	5.32	4.32
**2b**	12.06	7.24	6.38
**2c**	20.53	14.43	13.05
**2d**	20.52	18.51	12.95
**2e**	24.23	22.12	11.82
